# Breast milk vitamin B12 concentration and incidence of diarrhea and respiratory infections among infants in urban Tanzania: a prospective cohort study

**DOI:** 10.1186/s13104-020-05003-7

**Published:** 2020-03-18

**Authors:** Omar N. Lweno, Christopher R. Sudfeld, Ellen Hertzmark, Karim P. Manji, Anne Marie Darling, Wafaie W. Fawzi

**Affiliations:** 1 Ifakara Health Institute, Bagamoyo Research and Training Center, Bagamoyo, Tanzania; 2 Departments of Nutrition, Harvard T.H. Chan School of Public Health, Boston, MA USA; 3 Departments of Global Health and Population, Harvard T.H. Chan School of Public Health, Boston, MA USA; 4 Departments of Epidemiology, Harvard T.H. Chan School of Public Health, Boston, MA USA; 5 Muhimbili University of Health and Allied Sciences, Dar es Salaam, Tanzania

**Keywords:** Breast milk, Vitamin B12, Childhood infections

## Abstract

**Objective:**

A recent trial of vitamin B12 supplementation among Indian children 6–30 months found no effect on the incidence of diarrhea and respiratory infections. These results differ with studies in adults that showed improvement of the immune response following treatment with vitamin B12. We sought to determine how the adequacy of vitamin B12 concentrations in breast milk could act as immune modulator and protect against the incidence of diarrhea and respiratory infections of children up to 18 months in urban Tanzania.

**Results:**

A prospective cohort study was undertaken to determine the association of breast milk vitamin B12 concentration with the incidence of acute respiratory infection and diarrhea among infants in urban Tanzania. A random sample of 491 women enrolled in a trial of multivitamins provided milk for B12 analysis at or around 6 weeks postpartum. Of 491 women, 345 had breast milk vitamin B12 inadequacy (< 310 pmol/L). Using generalized estimating equations, we found no overall association of milk vitamin B12 concentration with incident diarrhea and acute respiratory infections in infants. Studies measuring longitudinal changes of breast milk B12 concentration over time are needed to clarify the role of breast milk vitamin B12 in childhood infections.

## Introduction

Vitamin B12 deficiency is common among women and infants in low and middle-income countries, partially due to inadequate intake and limited diet diversity [1]. Infants who develop vitamin B12 deficiency due to inadequate intake from breast milk or other sources may be at increased risk for infections, growth deficits, and neurodevelopmental delays [2]. A recent clinical trial in India reported that vitamin B12 supplementation among children 6–30 months of age had no effect on the incidence of diarrhea or acute lower respiratory tract infections [3].

Previous studies in adults have reported enhancement of cellular immune response following vitamin B12 supplementation [4] and restoration of both cellular and humoral immune responses following vitamin B12 treatment [5]. As a result, maternal breast milk vitamin B12 levels may be an infant immune modulator and determinant of infectious disease risk for breastfeeding infants.

We tested the hypothesis that greater concentration of vitamin B12 in breast milk is protective against the incidence of childhood diarrhea and respiratory infections in a cohort of mother and infant pairs in Dar es Salaam, Tanzania.

## Main text

### Methods

A random sample of 500 women who participated in a randomized trial of multivitamin supplementation [6] was selected for vitamin B12 breast milk analysis. Breast milk samples were collected at or around the 6th-week postpartum (mean = 5.4 weeks, SD = 0.5) visit between March 2003 and February 2005. They were stored at minus 80 degrees centigrade in a biobank at the Muhimbili University of Health and Allied Sciences (MUHAS). The material transfer agreement (MTA) between the MUHAS and the Harvard T.H. Chan School of Public Health (HSPH) was signed and approved by the chair of the medical research coordinating committee (MRCC) in Tanzania before the shipment of samples. Samples were shipped using dry ice from Tanzania to the USDA Western Human Nutrition Research Center in Davis, California for analysis by competitive chemiluminescent enzyme immunoassay [7]. Three samples were too small for analysis, four samples were collected more than 8 weeks after delivery, and two could not be linked to randomized study participants leaving 491 samples.

Breast milk vitamin B12 concentration at or above 310 pmol/L was considered to define adequacy [8].

Child morbidity was assessed once a month from birth to 18 months of age. Maternal reports about the occurrence of diarrhea and respiratory infections were recorded by study nurses during the monthly visit. The constellation of the following symptoms of cough, difficulties in breathing and any fever not exceeding 7 days was used to define acute respiratory infection whilst diarrhea was defined by the passage of more than three loose stools per day (24 h).

Log-binomial models were used to determine the relative risk of acute respiratory infection during follow-up comparing those with or without adequate vitamin B12 in their breast milk. Log-poisson models were used to compare incidence rates of diarrhea between those with adequate vitamin B12 levels in breast milk (reference group) and those with inadequate vitamin B12 leves in breast milk (exposed group). Additionally, we investigated the relationship between the risk of diarrhea and acute respiratory infection across the quartiles of vitamin B12 concentration in breast milk categorized as Q1 (less than 169 pmol/L), Q2 (169–205 pmol/L), Q3 (206–338 pmol/L), and Q4 (more than or equal to 339 pmol/L). Quartile 4 was used as a reference group in comparison to the other quartiles of breast milk vitamin B12 concentration. Generalized estimating equations (GEE) were used to account for repeated measurements of acute respiratory infection and diarrhea variables in infants. In multivariable models, all dichotomous and continuous covariates with *p* value less than 0.20 or categorical covariates with p-value for an individual level compared with a reference level less than 0.20 were used to fit a multivariable model. The resulting multivariable model for acute respiratory infection included child gender, birth weight, and maternal hemoglobin at 6 weeks postpartum. The multivariable model for diarrhea included treatment arm, maternal education, the Filmer-Pritchett wealth score, baseline BMI, and maternal hemoglobin at 6 weeks..

### Results

Characteristics of the study population are presented in Table 1. Out of the 491 women included in the study, 345 (70%) had less than adequate vitamin B12 in breast milk (< 310 pmol/L). Vitamin B12 concentration in breast milk less than adequate intake (< 310 pmol/L) at the 6th week postpartum was not associated with the risk of incident respiratory infection (RR = 1.01, 95% CI 0.90–1.14) or diarrheal disease (IRR = 0.95, 95% CI 0.86–1.05) in infants (Table 2). There was also no association of quartiles of vitamin B12 concentration in breast milk with the incidence of acute respiratory infection (p-values for trend > 0.05). The risk of diarrhea was lower among infants born to mothers whose breast milk vitamin B12 concentration was categorized in the first quartile (IRR = 0.85, 95% CI 0.75–0.97) and the third quartile (IRR = 0.95, 95% CI 0.84–1.07) but not the second quartile (IRR = 1.02, 95% CI 0.91–1.15) when compared to the reference group of fourth quartile (Table 2). However, the trend test was not statistically significant (p-values for trend > 0.05)..

**Table 1:** Descriptive characteristics of mothers by breastmilk vitamin B12 status at 6 weeks postpartum in Tanzania

Characteristic	Descriptive statistics	B12 < 310 pmol/L (N = 345)	B12 >=310 pmol/L (N = 146)
Treatment arm			
Placebo	N, column %	180 (52.2)	64 (43.8)
Multivitamins	N, column %	165 (47.8)	82 (56.2)
Maternal age in years	Mean, SD	25.9 (5.1)	25.5 (5.0)
Maternal age (years)			
< 20	N, column %	49 (16.3)	24 (18.3)
20–24	N, column %	89 (29.7)	36 (27.5)
25–29	N, column %	85 (28.3)	40 (30.5)
≥ 30	N, column %	75 (25.0)	29 (22.1)
Maternal education (years)			
0–4	N, column %	39 (11.4)	16 (11.1)
5–7	N, column %	219 (63.9)	91 (63.2)
8–11	N, column %	64 (18.7)	22 (15.3)
12+	N, column %	21 (6.1)	15 (10.4)
Maternal BMI at baseline (kg/m^2^)	Mean, SD	24.8 (4.1)	24.5 (4.1)
Maternal BMI categories (kg/m^2^)			
Less than 22.0	N, column %	77 (24.3)	43 (32.3)
22.0–24.9	N, column %	109 (34.4)	38 (28.6)
25.0–29.9	N, column %	101 (31.9)	37 (27.8)
30 or more	N, column %	30 (9.5)	15 (11.3)
Maternal Hb at 6 weeks (gm/dL)	Mean, SD	12.0 (1.6)	12.5 (1.8)
Maternal Hb categories at 6 weeks (gm/dL)			
Less than 8.5	N, column %	13 (3.8)	2 (1.4)
8.5–10.9	N, column %	58 (16.8)	20 (13.7)
11.0 or more	N, column %	269 (78.0)	121 (82.9)
Infant gender			
Female	N, column %	165 (47.8)	76 (52.4)
Male	N, column %	180 (52.2)	69 (47.6)
Birth weight (kg)	Mean, SD	3.2 (0.5)	3.1 (0.5)
Birth weight categories (kg)			
≤ 2.0	N, column %	7 (2.1)	6 (4.3)
2.001–2.499	N, column %	11 (3.3)	7 (5.0)
≥ 2.5	N, column %	320 (94.7)	127 (90.7)

**Table 2:** Breast milk vitamin B12 as a predictor of ARI and diarrhea among infants in Urban Tanzania

		Univariate analysis		Multivariate analysis^a^	
		Relative risk (95% CI)	P-value	Relative risk (95% CI)	P-value
Acute respiratory infection					
Breast milk vitamin B12 status					
Adequate intake (≥ 310 pmol/L)	146	Ref		Ref	
Less than adequate intake (< 310 pmol/L)	345	1.04 (0.93, 1.17)	0.49	1.01 (0.90, 1.14)	0.85
Quartiles of breast milk vitamin B12 (pmol/L)					
Q4 (more than or equal 339)	122	Ref	0.95	Ref	0.75
Q3 (206–338)	125	1.04 (0.90, 1.20)		1.01 (0.87, 1.17)	
Q2 (169–205)	127	0.95 (0.82, 1.11)		0.93 (0.80, 1.08)	
Q1 (less than 169)	116	1.04 (0.89, 1.20)		1.00 (0.86, 1.16)	

Diarrheal disease		Univariate analysis		Multivariate analysis (N = 471)^b^	
		Incidence rate ratio (95% CI)	P-value	Incidence rate ratio (95% CI)	p-value
Breast milk vitamin B12 status					
Adequate intake (≥ 310 pmol/L)	139	Ref		Ref	
Less than adequate intake (< 310 pmol/L)	325	0.99 (0.90, 1.08)	0.77	0.95 (0.86, 1.05)	0.30
Quartiles of Breast milk vitamin B12 (pmol/L)					
Q4 (more than or equal 339)	117	Ref	0.54	Ref	0.06
Q3 (206–338)	115	0.94 (0.83, 1.06)		0.95 (0.84, 1.07)	
Q2 (169–205)	123	1.07 (0.95, 1.21)		1.02 (0.91, 1.15)	
Q1 (less than 169)	109	0.91 (0.80, 1.03)		0.85 (0.75, 0.97)	

Estimates and p-values based on generalized estimating equations (GEE) with the log link and the binomial distribution, with a working exchangeable correlation structure

^a^Multivariable model include child sex, birth weight and linear maternal hemoglobin at 6 weeks postpartum

^b^Multivariable model include maternal treatment arm, maternal education, maternal wealth score, baseline BMI, and linear maternal hemoglobin at 6 weeks postpartum. Estimates and p-values based on generalized estimating equations (GEE) with the log link and the Poisson distribution, with an independent correlation structure

### Discussion

Our study found a high proportion of women (70%) with less than adequate concentration of vitamin B12 in breast milk in urban Tanzania. It was slightly lower than that reported in a study conducted in Kenya (89%) where breast milk samples were collected at 6 months postpartum and Cambodia (75%) where breast milk was collected at 12 months postpartum and the cut-off value to define adequacy was 365 pmol/L [8, 9].

In this study, we found no overall association of breast milk vitamin B12 status with incident diarrhea and acute respiratory infections both as a binary exposure (adequate and inadequate) or expressed as quartiles. Our results are similar to results from India by Strand and colleagues that found no association of infant vitamin B12 deficiency with incidence of respiratory tract infection [10].

Our results are similar to a cross-sectional study in Nepal that reported a high prevalence of vitamin B12 deficiency among children with acute diarrhea enrolled in a zinc supplementation trial [11]. They found no significant association of child plasma cobalamin and folate with the duration and numbers of loose or watery stool although the use of plasma samples limit direct comparison to breast milk samples. We cannot attribute causality of acute diarrhea to cobalamin or folate deficiency because of the cross-sectional design of the Nepal study, however, it highlights the need of considering how plasma levels of the two micronutrients are affected by intake from breast milk and other dietary sources during infancy. Clinical studies that measure absorption of vitamin B12 from breast milk are required to better understand its effect on plasma vitamin B12 and the status of its functional biomarkers.

### Conclusion

Overall, the proportion of women with concentrations indicating inadequate vitamin B12 in their breast milk is high in urban Tanzania. We found no association of vitamin B12 concentration in breast milk with incidence of diarrhea and respiratory infections among Tanzanian infants. Prospective studies that measure the longitudinal variation of breast milk B12 concentration and the incidence of childhood infections are needed to clarify its role in childhood infections.

## Limitations

Our study had several limitations. First, we assessed vitamin B12 concentration at a single time-point which does not allow for assessment of changes in breast milk composition over time. In addition, we did not have information on the concentrations of other micronutrients in breast milk [12], maternal antibodies, the frequency of breastfeeding and gut microbiota [13] which may also influence the incidence of diarrhea and respiratory infections. Other residual confounders that should be investigated in future studies of micronutrient concentration and adequacy in breast milk include breast milk volume, infant micronutrient intake from breast milk, infant weight, infant age, parity, and micronutrient status of the mothers during pregnancy [14]. Further, no information was available on diarrhea severity, which may modify its relationship to B12 status [15]. In addition, we measured incidence of diarrhea and respiratory infections using maternal report which may have led to some degree of misclassification.

## Data Availability

The datasets used and analyzed during the current study are not publicly available. However, the datasets are available from the authors upon reasonable request and with permission of sponsor.

## Abbreviations

AI: Adequate intake
BMI: Body mass index
CI: Confidence interval
GEE: Generalized estimating equations
GID: Global infectious disease
Hb: Hemoglobin
HSPH: Harvard T.H. Chan School of Public Health
IRB: Institutional review board
Kg: Kilogram
PNS: Perinatal study
MRCC: Medical Research Coordinating Committee
MTA: Material Transfer Agreement
MUHAS: Muhimbili University of Health and Allied Sciences
NatHREC: National Health Research Ethics Committee
NIH: National Institute of Health
NIMR: National Institute for Medical Research
RR: Relative risk
USDA: United States Department of Agriculture
